# A high-performance compilation strategy for multiplexing quantum control architecture

**DOI:** 10.1038/s41598-022-11154-3

**Published:** 2022-05-03

**Authors:** Zheng Shan, Yu Zhu, Bo Zhao

**Affiliations:** 1 State Key Laboratory of Mathematical Engineering and Advanced Computing, Zhengzhou, 450001 Henan China; 2 Songshan Laboratory, Zhengzhou, 450001 Henan China

**Keywords:** Computer science, Quantum information

## Abstract

Quantum computers have already shown significant potential to solve specific problems more efficiently than conventional supercomputers. A major challenge towards noisy intermediate-scale quantum computing is characterizing and reducing the various control costs. Quantum programming describes the process of quantum computation as a sequence, whose elements are selected from a finite set of universal quantum gates. Quantum compilation translates quantum programs to ordered pulses to the quantum control devices subsequently and quantum compilation optimization provides a high-level solution to reduce the control cost efficiently. Here, we propose a high-performance compilation strategy for multiplexing quantum control architecture. For representative benchmarks, the utilization efficiency of control devices increased by 49.44% on average in our work, with an acceptable circuit depth expansion executing on several real superconducting quantum computers of IBM.

## Introduction

Benefit from the natural features of quantum superposition and quantum entanglement, quantum computing (QC) has significant potential for solving classically intractable computational problems in areas such as machine learning^1^, cryptography^2^, chemistry^3,4^ and others. There are various quantum computing physical implementation systems up to now. Superconducting quantum computing has steady progress in gate and measurement fidelity^5–8^ and IBM’s roadmap for scaling quantum technology^9^ has been verified solidly so far^10^ based on this technical route. However, the implementation of a practical architecture that can scale to millions of qubits and solve large-scale practical problems remains a challenging milestone^11^. Actually, superconducting quantum processors must be placed in a cryogenic environment to be initialized close to their quantum ground state. To control its quantum state evolution, each qubit is individually addressed with corresponding microwave signal lines connected to room temperature control devices. The control costs and complexity related to the multiple coaxial lines per qubit limit the possible scalable size of a superconducting quantum chip, whose upper limit is about a few thousand qubits subjected to current techniques^11^.

Currently, physical operations, such as pulsed excitation, are used to conduct quantum computation in the hardware level. These physical operations are normally described by unitary operators that show the state evolution of qubits. Intuitively, quantum computation can be regarded as circuits of quantum gates with a series of ordered sequences of unitary operators^12^. However, gate-model quantum computers have inherent constraints in their architecture including coupling graph, primitive gates supported^13–15^, etc. Quantum circuits cannot be directly executed on these computers. According to the Solovay Kitaev theorem, we can approximate any quantum unitary operations as quantum circuits based on a finite set of operators within an arbitrary tolerance. However, finding an optimal strategy to build and compute such a sequence remains in suspense. The problem is defined as quantum compilation and optimization to rebuild the approximating optimal circuits suitable for different platforms. Quantum circuit compilation (QCC) takes a nonconforming quantum circuit as input and generates a circuit that can be executed on the target platform according to its constraints, including coupling graph, primitive gate set and so on. A quantum compiler is a toolchain between quantum program and pulse scheduling, with which programmers do not need to consider the physical constraints of a particular quantum processor. Every quantum compiler has its own trade-off between the number of qubits, the length of the sequences, the compilation time and the final execution time.

Generally, the quantum hardware can be modeled in four abstract layers: the quantum data plane where the qubits reside, the control and measurement plane, the control processor plane, and the host processor^16^. Challenges at the quantum-classical interface are examined with the goal of architecting a scaled-up quantum computer comprising millions of qubits. The quantum-classical interface, consisting of control and readout sub-systems, connects platform-specific operation to high-level software tools. These sub-systems include digital logic for generating and detecting analog waveforms, signal sources, data converters, amplifiers and so on. Separating the distinct sub-systems of the interface that perform readout and control, general arguments are given for why distributing the components of these sub-systems over significant distances and across large temperature gradients presents a major challenge to scaling-up the technology^17^. Largely addressing these issues, an architecture for the interface that leverages cryo-CMOS circuits proximal to the quantum plane is motivated, leveraging protocols that enable massively-parallel readout of qubits via frequency multiplexing^17,18^.

The novelty of this paper is performing a significant control optimization at software level in quantum computing. Our work targets the multiplexing quantum control architecture with M control channels and N qubits, where $2\le M\le N$. Theoretically, all high-level quantum logic gates can be decomposed into the combination of basic single qubit operations and two qubits controlled operations. Consequently, the extreme case of our quantum control architecture has two centralized control channels, controlling each qubit through a multiplexer. The experiment results show that our work can reduce the hardware cost of control system greatly and improve its utilization efficiency significantly while introducing limited circuit depth expansion.

## Results

### High-efficiency control architecture

**Figure 1:**
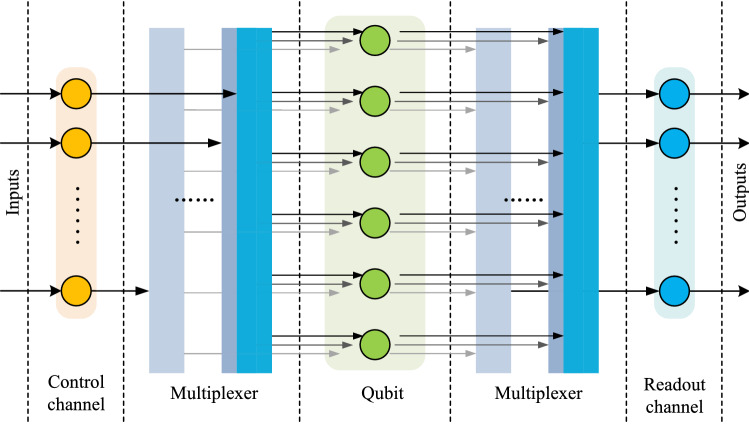
Centralized control and multiple readout system for superconducting quantum computing. There are *M* control channels, *N* qubits and *K* readout channels, where $2 \le M \le N$ and $1 \le K \le N$.

Considering the parallelism and quantum circuit depth, it is better to use one control channel to control a qubit respectively. However, the utilization efficiency of the quantum control system is limited when running quantum benchmarks. We do experiments on several quantum platforms of IBM with one-to-one control system. Based on the experimental results, we calculate the utilization efficiency of control systems during execution. The utilization efficiency is the ratio of the time when control channels are not idle to the whole execution time period. The average utilization efficiency of the benchmarks is about 60% in our experiments. To further improve the utilization efficiency and reduce the number and costs of control channels, we use *M* control channels to control *N* qubits, losing a little parallelism accordingly, as shown in Fig. 1. Obviously, $M \le N$ and the extreme case is $M = 2$ because of the existence of two-qubits operations.

### Dependence analysis

Some of the control operations are independent such as different single qubit operations applied to independent qubits. In addition, there exists a number of operations which have dependences to each other. Here, we define the dependences in quantum control system as two main kinds, sequence dependence and coherence dependence.

*Sequence dependence* is defined as the dependence between two control operations with timing sequence. These two operations are used on the same single qubit or two qubits with orders of priority. For example, a controlled-NOT (*CNOT*) gate on two qubits $q_i$ and $q_j$, denoted as $CNOT(q_i,q_j)$, can be executed only when all its predecessor gates on $q_i$ and $q_j$ have been executed already.

*Coherence dependence* is defined as the dependence between two qubits with controlled operations, such as CNOT. These two qubits are tightly coupled and should be controlled in the same time slots.

The two kinds of dependences above are both true dependences and cannot be eliminated at the compiler level. Related quantum operations should be executed orderly or simultaneously. Consequently, our high-efficiency quantum control architecture has at least two control channels to deal with the coherence dependence.

### High-performance compilation strategy

**Figure 2:**
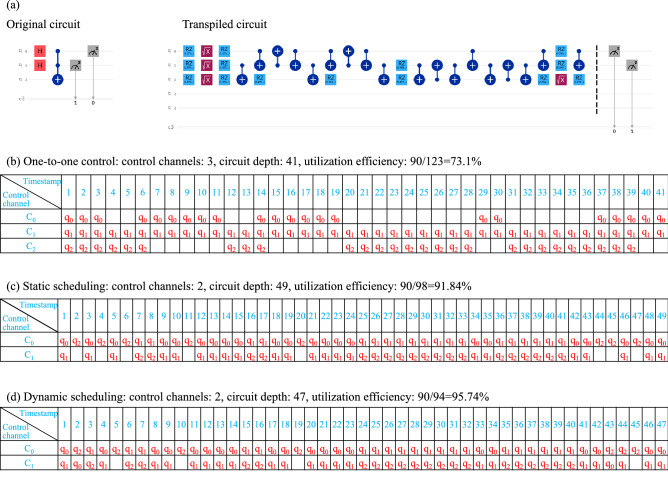
(**a**) The decomposed result of the original quantum circuit. (**b**) The scheduling sequence of traditional one-to-one control method. (**c,d**) The optimized results using static scheduling and dynamic scheduling, respectively.

To fully exploit the advantages of the multiplexing control architecture, we optimize the quantum circuit at basis gates level. Considering a Toffoli gate, it will be decomposed into a sequential circuit consisting of a series of single-qubit and two-qubit operations. Figure 2 gives a simple example with two Hadamard gates and a Toffoli gate and Fig. 2a shows the decomposed circuit. Obviously, traditional one-to-one control method has a higher efficiency when considering circuit depth. However, the overall utilization efficiency of the control system is dissatisfactory. The cost of control system plays an important role in NISQ era and quantum error correction and should be taken into consideration carefully when balancing the budget and overall performance. Figure 2b shows the control process and results.

A scalable quantum device with a multiplexing control architecture could be a solution to save the cost of control system. As shown in Fig. 1, there are limited control channels connected to each qubit through a multiplexer. Due to the limited control channels, some of the gates cannot be executed in parallel as in traditional compilation. The quantum circuit should be rescheduled during compilation to guarantee all the gates being executed in a right order. Here we propose two scheduling methods for multiplexing quantum control devices. The static scheduling method simply gives a control order of gates that could have been executed in parallel in traditional one-to-one control system. To further optimize the circuit, a dynamic scheduling method is given based on dependency analysis.

*Static scheduling.* A basic quantum circuit is obtained after transpilation and optimization. Without loss of generality, we assume a single-qubit quantum operation is executed in one time slot and a two-qubits quantum operation is executed in two time slots. Based on the assumption, we can get a basic quantum circuit with timestamps. Taking a quantum control system with two control channels as an example, we use $c_0$ and $c_1$ to indicate the control channels and $t_0, t_1, ..., t_s$ to represent the timestamps. For static scheduling, we use the decomposed circuit directly to map the control channels without any timing optimization, as shown in Fig. 2c. This method is simple and can guarantee the validity of the compiler easily. To further improve the performance, we bring about a dynamic scheduling scheme with timing optimization.

*Dynamic scheduling.* To further improve the utilization efficiency of quantum control channels and reduce the circuit depth, we propose a dynamic scheduling algorithm based on dependence analysis. A timing optimization is applied first and the independent quantum operations are reordered to maximize the utilization efficiency of control channels. The independent control operations can be reordered dynamically and spatiotemporally. We assume the control channels as hardwares of a pipelining and control operation to qubits as instructions. Our dynamic scheduling method is similar to the out-of-order launch and sequential execution process in classical computing. The result of dynamic scheduling is shown in Fig. 2d. The practically executed circuit depth can be further reduced by 4.1% and the utilization efficiency improved by 4.2%.

**Figure 3:**
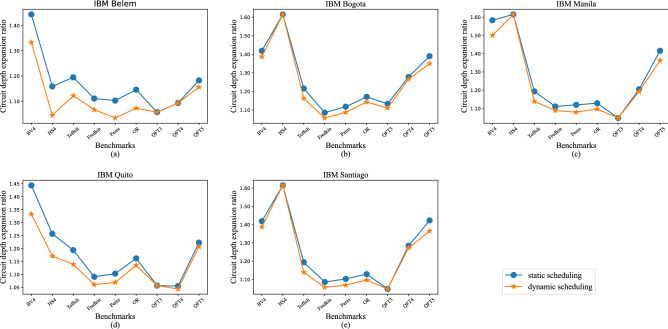
Circuit depth expansion based on various IBM quantum computers. The baseline is the circuit depth using a traditional one-to-one control system. The orange line with star marker shows the expansion ratio using our self-defined static scheduling method and the blue line with circle marker shows the results using our dynamic scheduling method inspired by scoreboard algorithm in classical computing.

**Figure 4:**
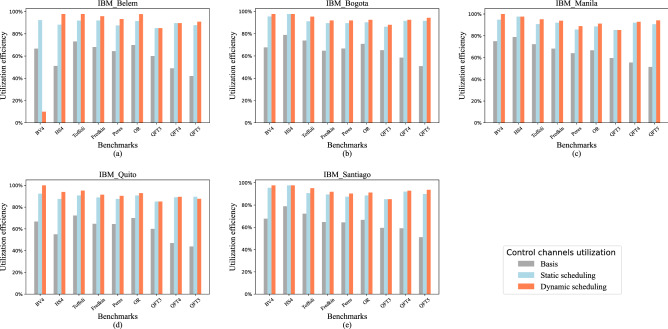
Utilization efficiency of control channels on various IBM quantum computers. The baseline is the utilization efficiency using traditional one-to-one control system. The other two show the results using static scheduling method and dynamic scheduling method respectively.

*Experimental results evaluation.* To validate the advantages of our scheme, we performed experiments on five IBM quantum computers with various benchmarks, including the Bernstein–Vazirani algorithm (BV)^19^, hidden shift algorithm (HS)^20,21^, quantum Fourier transform (QFT)^22^ and multi-qubit QC gates such as Toffoli and Fredkin^23^. We use these benchmarks because they constitute an essential part of many large QC applications that have been used in prior work on NISQ system evaluation^24–28^. The details of the benchmark are listed in Table 1. According to the experimental results shown in Figs. 3 and 4, we can improve the utilization efficiency significantly while only introducing a little circuit depth expansion. In spite of the hardware differences, the trends of optimization and extra cost are similar. The utilization efficiencies of traditional quantum control systems based on various benchmarks are ranging from around 50–80%. Our static scheduling method can help to improve the range from 90 to 100%. Compared with static scheduling, our dynamic scheduling method can bring about an extra 4% performance improvement. Our high-performance compilation strategy for multiplexing control quantum architecture has significant potential to implement quantum algorithms which need relatively fewer qubits while deeper circuit depth, such as Grover algorithm. The circuit depth expansion and utilization efficiency of control channels after scheduling depend on the number of channels and the quantum circuits. Before scheduling, the circuit needs to be compiled adapting to the hardware constraints. Due to different hardware constraints including coupling graph, the transformed circuit before scheduling varies on different platforms, which leads to different results on different devices. On IBM Belem, the utilization efficiency of QFT5 increased from 42.02 to 90.87% (increased by 116.25%). The utilization efficiency of the nine benchmarks increased by 60.16% on average on IBM Belem. On IBM Bogota, the average utilization efficiency increased by 42.85%. The values were 44.06%, 55.48% and 44.67% on the other three platforms. In summary, the utilization efficiency increased by 49.44% on average. Some of the benchmarks like QFT3 has a good result with high utilization efficiency and low circuit depth expansion. Restricted to the dependence among gates, few gates in circuit QFT3 can be executed in parallel even in one-to-one control system. When we decrease the number of control channels, the parallelism of the circuit will not be affected much. So we can get a low circuit depth expansion after scheduling in QFT3. The results show a trade-off between depth expansion and utilization efficiency. In general, with the increase of *M*, both depth expansion and utilization efficiency will decline.

**Table 1:** Benchmarks used in this paper.

Benchmarks	Qubits	Description
BV4	4	Quantum algorithm for determining the mathematical function g quantum oracle function, which is a black box operator which gives a dot product of a secret string
HS4	4	Quantum algorithm for the generalized hidden shift problem
Toffoli	3	A control-flip multi-qubit gate
Fredkin	3	A control-swap multi-qubit gate
Peres	3	A quantum circuit function that can compute the Peres gate, which is a basic reversible logic gate used in various reversible circuit
OR	3	A quantum circuit function that can compute the OR gate
QFT3	3	The quantum implementation of the discrete Fourier transform with 3 qubits
QFT4	4	The quantum implementation of the discrete Fourier transform with 4 qubits
QFT5	5	The quantum implementation of the discrete Fourier transform with 5 qubits

## Discussion

In this paper, we proposed a high-performance compilation strategy for multiplexing quantum control architecture. The experimental results showed the efficiency of our strategy. The quantum circuits of complex quantum algorithms can be decomposed and transpiled into basic quantum circuits matching target quantum instruction sets. For future work, we would focus more on the trade-off between depth expansion and utilization efficiency when M changes and study how to choose M to get a satisfactory result. We will also try to apply our strategy to quantum algorithms solving practical problems based on larger-scale quantum devices. In addition, we are going to pay more attention to new mapping and compilation methods to further improve the performance of our scheduling schemes.

## Methods

In this section, we introduce our optimization strategies for multiplexing quantum control architecture. We first give preprocessing steps in the "Preprocessing" section. The static scheduling and dynamic scheduling methods are introduced in the "Static scheduling and dynamic scheduling" section. Then we analyse complexity of our methods in the "Complexity analysis" section. We summarize the notations we use in Table 2.

**Table 2:** Definition of notations.

Notation	Definition
*M*	Number of control channels
*N*	Number of physical qubits
*n*	Number of logical qubits in quantum circuit
*g*	Number of gates in quantum circuit
$q_{\{0,1,\ldots ,n\}}$	Logical qubits in quantum circuit
*DAG*	The execution constraints of gates in quantum circuit, defined in the "Preprocessing" section
*DAGT*	Circuit DAG with timestamp, defined in the "Preprocessing" section
*F*	Front layer, defined in the "Preprocessing" section

### Preprocessing

Before applying our methods, some preprocessing steps need to be done to generate required data including circuit DAG, initialized circuit DAGT and initialized front layer.

*Circuit DAG*. The sequence dependences of gates can be abstracted by a circuit Directed Acyclic Graph (DAG), where the gates are the vertices, and the edges are the execution dependences of gates. A two-qubit gate $CNOT(q_i,q_j)$ can be executed only when all its predecessors on $q_i$ and $q_j$ have been executed already. A DAG can be constructed by traversing the entire quantum circuit with complexity *O*(*g*), where *g* is the number of gates in circuit.

*Circuit DAGT*. We define circuit directed acyclic graph with timestamps (DAGT) by assigning each vertex of circuit DAG with a timestamp. The timestamp of a gate is the earliest time it can be executed. Gates with the same timestamp can be executed at the same time. The gates in circuit are classified in single-qubit gates and two-qubit gates. Without loss of generality, here we assume the duration time of single-qubit gate is 1 timeslot while two-qubit gate is 2 timeslots.

*Front layer*. A front layer is a set of gates whose predecessors have all been executed. It can be initialized by choosing the vertices in circuit DAGT whose indegree is zero. These vertices represent for the gates that do not need to be executed after any other gates in quantum circuit.

Algorithm  1 shows the pseudo code of our algorithm for generating a DAGT for one-to-one control system by scanning through the entire DAG. In the beginning, the circuit DAGT is initialized with the circuit DAG by assigning each vertex with timestamp 0. Front layer *F* needs to be initialized, too. In each iteration, the algorithm will first check whether all the gates in *F* are executable (whether the timestamp of gate is equal to the current timestamp *ts*). For each executable gate, we obtain its successor gates and update their timestamps. A successor gate’s timestamp is the maximum of the sum of its predecessor’s timestamp and the duration time. If a successor gate’s dependencies are all resolved (all its predecessors are removed from *F*), it can be added to *F*. The algorithm terminates when all gates are executed.

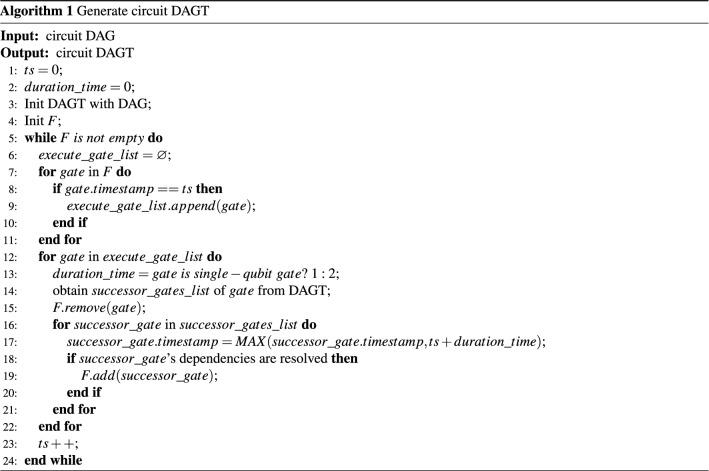



### Static scheduling and dynamic scheduling

In the "Preprocessing" section, the timestamps of gates in circuit DAGT are computed considering the sequence dependence constraint and different duration time of gates. However, on the platform with multiplexing control architecture, there are also control constraints. As shown in Fig. 1, there are limited control channels connected to each qubit through a multiplexer with which qubits can be controlled through time division multiplexing. Due to the limited control channels, some of the gates cannot be executed in parallel. The quantum circuit should be rescheduled to guarantee all the gates being executed in a right order.

Algorithm 4 shows the pseudocode of a static scheduling method to generate a circuit DAGT for multiplexing control architecture. Algorithm 4 first initializes DAGT, *F* and *successors*. The variable *ts* is the current timestamp. To represent the information about control channels, we use $channels\_unoccupied$ to represent the number of idle channels and a *channelList* to record the remaining time to be occupied of each channel. A gate is executable only when it is in *F* and there are enough idle channels left. In the iteration, the algorithm will check whether all the gates in *F* are executable. If a gate is executable, it will find idle channels for it and record the remaining occupied time of the channel. Then the algorithm will obtain successors of this gate in DAGT and add them to *successors*. If there are not enough idle channels, it will increase timestamp and update the remaining occupied time of channels. When all the gates in *F* are executed, the algorithm will assign *F* with *successors* and start iteration again until there are no successors. Algorithms 2 and 3 give the pseudocode of allocating and updating control channels.

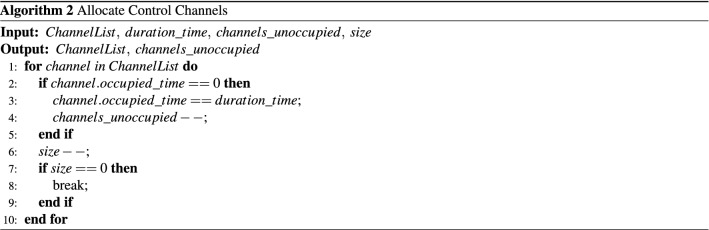

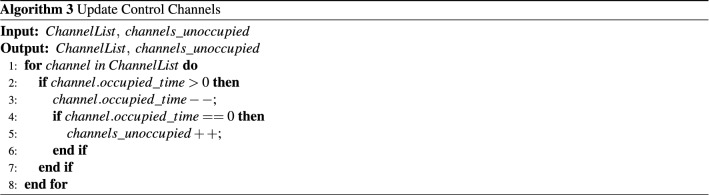





Although Algorithm 4 gives a scheduling method for multiplexing control architecture, there still remains room to reduce the circuit depth and improve the utilization efficiency of control system. In Algorithm  4, only when all gates in *F* have been executed completely can the successors be added to *F*. However, some of the successors whose dependences are all resolved can be executed once there are enough idle control channels. In this way, the utilization efficiency of control system can be improved. The circuit depth may also be reduced than static scheduling. Thus, we propose a dynamic scheduling method based on the static scheduling. Algorithm 5 shows the pseudocode. Different from static scheduling, Algorithm 5 will add new gates to *F* dynamically if possible.

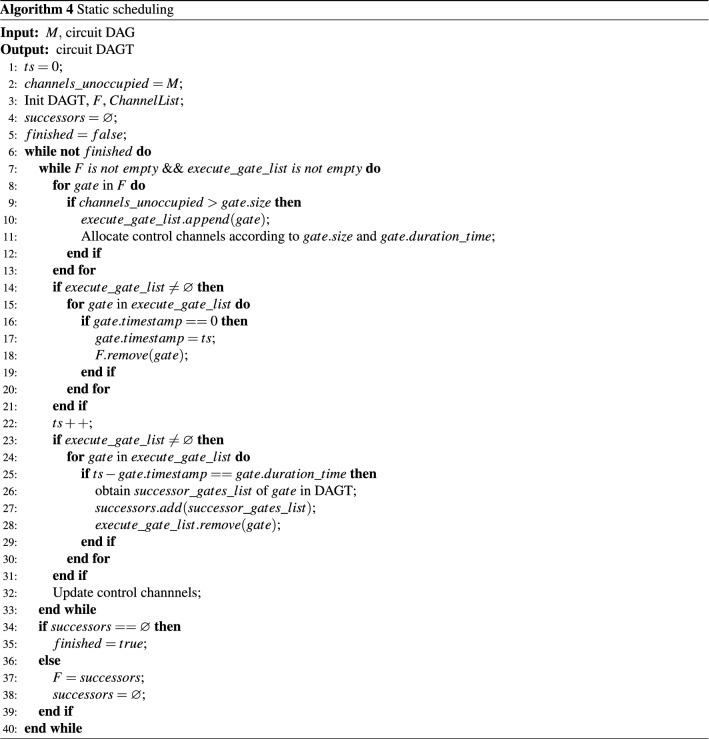

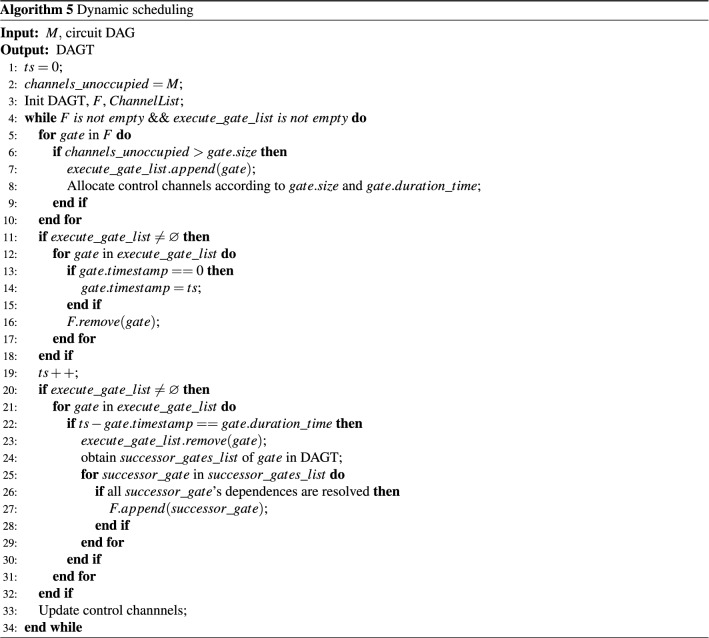





### Complexity analysis

In Algorithm 1, the circuit DAGT can be generated by traveling the circuit DAG. The complexity of Algorithm 1 is $O(g+e)$ where *g* is the number of gates and *e* is the number of edges of circuit DAG.

Algorithm 4 can be divided into four steps: find executable gates in *F* and allocate channels, assign timestamps for executable gates and increase timestamps by one, remove gates that finish executing and add its successors whose dependencies are all resolved, update channels. Each gate in the circuit will be executed once in the algorithm. Both Allocating channels (Algorithm 2) and Updating channels (Algorithm 3) have a complexity of *O*(*M*). The worst case is that only one gate can be executed in each iteration. In this case, the complexity of Algorithm 4 is $O(g\times M+e)$, which has more relations with circuit size and channel numbers. *M* is much less than the number of qubits in multiplexing control quantum architecture. Algorithm 5 has the same complexity as Algorithm 4, which is acceptable in compilation.
